# MicroRNA-155 Promotes Glioma Cell Proliferation via the Regulation of MXI1

**DOI:** 10.1371/journal.pone.0083055

**Published:** 2013-12-23

**Authors:** Jianwen Zhou, Wei Wang, Zhenhua Gao, Xueling Peng, Xulin Chen, Wei Chen, Weiyi Xu, Haixiong Xu, Marie C. Lin, Songshan Jiang

**Affiliations:** 1 State Key Laboratory of Biocontrol, School of Life Sciences, Sun Yat-sen University, Guangzhou, China; 2 Key Laboratory of Gene Engineering of the Ministry of Education, School of Life Sciences, Sun Yat-sen University, Guangzhou, China; 3 Neurosurgery Department, Epilepsy Centre, Guangzhou General Hospital, Guangzhou Command, PLA, Guangzhou, China; 4 Department of Radiology, First Affiliated Hospital, Sun Yat-sen University, Guangzhou, China; 5 Department of Gynecology, the Second Affiliated Hospital of Guangzhou Medical University, Guangzhou, China; 6 Department of Neurosurgery, Affiliated Shantou Hospital of Sun Yat-sen University, Shantou, China; 7 Biomedical Eng. Res. Center, Kunming Medical University, Kunming, China; 8 Department of Pathology, First Affiliated Hospital, Sun Yat-sen University, Guangzhou, China; 9 HuaBo Bio-Pharmaceutic Institute of Guangzhou, Guangzhou, China; 10 Shenzhen State High-Tech Industrial Innovation Centre, Shenzhen, China; NIH/NCI, United States of America

## Abstract

Gliomas are the most common and aggressive primary tumors in the central nervous system. Recently, Max interactor-1 (MXI1), an antagonist of c-Myc that is involved in brain tumor progression, has been reported to be deregulated in a variety of tumors including glioma. However, the mechanism of MXI1 deregulation in gliomas remains unclear. In this study, we show that the relative expression level of MXI1 is markedly down-regulated in glioma cell lines. Using integrated bioinformatic analysis and experimental confirmation, we identified several miRNAs by screening a panel of predicted miRNAs that may regulate the MXI1 3′UTR. The strongest inhibitory miRNA, miR-155, can attenuate the activity of a luciferase reporter gene that is fused with the MXI1 3′UTR and decrease the expression levels of MXI1 mRNA and protein in U87 glioma cells. The potential role of miR-155 in promoting glioma cell proliferation by targeting MXI1 was confirmed in various glioma cell lines by rescue experiments using MTT assays, EdU incorporation assay, and cell counting experiments. In addition, we determined that the level of MXI1 mRNA was inversely correlated with the expression of miR-155 in 18 sets of glioblastoma multiforme specimens. These findings reveal for the first time that the targeting of MXI1 by miR-155 may result in a reduction in MXI1 expression and promote glioma cell proliferation; this result suggests a novel function of miR-155 in targeting MXI1 in glioma-genesis.

## Introduction

Gliomas are the most common and aggressive primary tumors in the central nervous system; the average survival for glioblastoma patients is only 14 months [1]. There have been advancements in surgery, radiation and medical therapies for the treatment of glioblastoma, but the etiology of the disease is largely unknown [2]. Hence, it is crucial to identify the critical carcinogenic pathways and identify new and effective therapeutic targets for this devastating disease.

MXI1 is a member of the Mad family of transcription factors that counteracts the activity of c-Myc, which activates transcription and promotes cell proliferation by competing with Max and by recruiting the Sin3 transcriptional repressor [3], [4]. MXI1 can also directly repress the transcriptional activity of the c-Myc promoter [5]. Knockout experiments in mice have confirmed the tumor suppressor role of MXI1 [6]. MXI1 is located at 10q24-25 [7], [8] a region where loss of heterozygosity (LOH) has been reported to occur in several human cancers, including prostate tumors, renal cell carcinomas, meningiomas, endometrial cancers, small-cell lung cancers and gliomas [9]. Several studies have reported MXI1 mutations in prostate tumor specimens [10], [11], but these mutations appeared to be rare in both prostate tumors [12], [13] and gliomas [14], [15]. It has also been reported that the MXI1 gene is often expressed at a low level in testicular tumors [16].

Wechsler et al's work showed that MXI1 suppresses human glioma cell growth [14]; in the presence of normal levels of c-Myc, the inactivation of the MXI1 gene enhances proliferation and inhibits differentiation. Consistent with this, in the G2/M phase, the overexpression of MXI1 promotes the differentiation of glioma cells and decreases the cell proliferation via repressing the cyclin B1 gene expression during transcription [17]. Therefore, it could be predicted that in certain tumors, the loss of MXIl function may lead to tumor progression [14]. Based on these studies, we hypothesized that the down-regulation of MXI1 may lead to the acceleration of cell proliferation. However, the molecular mechanism of MXI1 down-regulation is still unclear. Accumulating evidence suggests that microRNAs (miRNAs) are involved in the process of glioma formation and growth [1]. miRNAs regulate gene expression primarily via their interaction with the 3′UTRs of target mRNAs, resulting in mRNA decay or translational repression [18], [19]. Therefore, we speculated that some miRNAs may be responsible for the low expression of MXI1 in gliomas.

In this study, we demonstrated that the expression level of MXI1 was very low in glioma cell lines. By computational prediction and experimental confirmation, we identified miR-155 as one miRNA that directly targets MXI1 and down-regulates MXI1 mRNA and protein level. miR-155 is an oncogenic miRNA encoded by an exon of the noncoding RNA known as the B-cell integration cluster (BIC) [20], which is located on chromosome 21, was originally identified as a common retroviral integration site for the avian leukosis virus, and has been found to be transcriptionally activated in B-cell lymphomas [21]–[24]; we therefore investigated the role of miR-155 in promoting the proliferation of glioma cells. Furthermore, we determined the expression levels of MXI1 and miR-155 in 18 sets of glioblastoma multiforme specimens and paired normal tissue specimens. Additionally, we demonstrated that the level of MXI1 mRNA is inversely correlated with miR-155 expression. Together, these results indicate that miR-155 promotes glioma cell proliferation partially by down-regulating the expression of MXI1; this result suggests that MXI1 could be a new functional target of miR-155 in glioma formation.

## Materials and Methods

### Vector construction

To express miRNAs, human genomic fragments containing miRNA precursors (pre-miRNAs) with 80 to 150 bp of flanking sequences on both sides were amplified and cloned into the modified pLL3.7 vector under the control of the human U6 promoter. The synthesized oligonucleotides used for pre-miRNA cloning are listed in Table S1. The full-length 3′UTR of MXI1 and the first and second halves of the MXI1 3′UTR were cloned downstream of the *Renilla* luciferase reporter gene in the psiCHECK-2 vector (Promega, Madison, WI, USA). Mutations in the seed region of miR-155 binding sites were introduced into MXI1 luciferase reporters using overlap PCR. Human MXI1 cDNA without its native 3′UTR was cloned into the XhoI and BamHI sites downstream of the CMV promoter in the cDNA expression vector pcDNA-neo, which was modified from pcDNA3.1. These constructs were named pLL3.7-miRNAs, MXI1-3′UTR, MXI1-3′UTR1, MXI1-3′UTR2, MXI1-3′UTR1 mut, MXI1-3′UTR2 mut, and MXI1 cDNA. The primers employed above were as follows: MXI1-3′UTR F: 5′-CTCGAGTAGAACCCAGCATGACATAACAGTG-3′, MXI1-3′UTR R: 5′-GGATCCTTCTTCGTTCACAGTTTTTATTTCTTC-3′; MXI1-3′UTR1 F: 5′-CCGCTCGAGGACATAACAGTGCAGGGCAAAATA-3′, MXI1-3′UTR1 R: 5′-CGGGATCCAAACAGCCAGGGGTAAGGTCTC-3′; MXI1-3′UTR2 F: 5′-CCGCTCGAGATTGATAGATCTTTATGTTTAGATAGGGCTGGGCAAG-3′, MXI1-3′UTR2 R: 5′-CGGGATCCTCTTCGTTCACAGTTTTTATTTCTTC-3′; MXI1-3′UTR1-mut F: 5′-ATTTTGATGCTCGTAATGATAATGATAAAACACCTC-3′, MXI1-3′UTR1-mut R: 5′-TTTATCATTATCTTACGAGCATCAAAATAGATGATT-3′; MXI1-3′UTR2-mut F: 5′-GCCTTCGCTTCGUAATATTGGGCCTTCATTCAGATGA-3′, MXI1-3′UTR2-mut R: 5′-ATGAAGGCCCAATATTACGAAGCGAAGGCCATGAGAA-3′; MXI1 cDNA F: 5′-CACAACTCGAGATGGAGCGGGTGAAGATGATCAAC-3′, and MXI1 cDNA R: 5′-AAGGATCCTGCACTGTTATGTCATGCTGGGT-3′. All the constructs were confirmed by DNA sequencing.

### Cell lines, patient specimens, cell culture and cell transfection

HEK-293T (293T) cells and the human glioma cell lines U87, U251 and A172 were obtained from the American Type Culture Collection (Manassas, VA, USA) and maintained in a 37°C, 5% CO_2_ incubator in DMEM supplemented with 10% fetal bovine serum (FBS) and penicillin/streptomycin (100 U/ml). Brain normal tissues, glioblastoma multiforme specimens (GBM, WHO IV) and their corresponding normal adjacent tissues were obtained during surgery from patients at the Affiliated Shantou Hospital of Sun Yat-sen University (Shantou Central Hospital). All of the samples were obtained with patients' written informed consent and were histologically confirmed. The study was approved by the Ethics Committee of Shantou Central Hospital.

The miR-155 mimic, the miR-155 inhibitor, and their cognate control RNAs were synthesized and purified by GenePharma, Shanghai, China. The RNA sequences mentioned above were as follows: miR-155 mimic: sense 5′ UUAAUGCUAAUCGUGAUAGGGTT 3′ and antisense: 5′ CCCUAUCACGAUUAGCAUUAATT 3′. Mimic control: sense 5′ UUGUCCGAACGUGUCACGUTT 3′ and antisense: 5′ ACGUGACACGUUCGGAGAATT 3′. miR-155 inhibitor: 5′ ACCCCUAUCACGAUUAGCAUUAA 3′. Inhibitor control: 5′ CAGUACUUUUGUGUAGUACAA 3′. The control RNAs contain random sequences that are not predicted to have any interactions in cells. Cell transfection was performed using Lipofectamine 2000 (Invitrogen, Carlsbad, CA, USA) according to the manufacturer's instructions. To transfect RNA oligonucleotides, 100 nmol/L of miRNA mimic or antisense oligonucleotides were used. The transfection efficiency was estimated to be approximately 80% for U87, U251 and A172 cells by using Cy3 dye-labeled RNA oligonucleotides (Ribobio, Guangzhou, China). Plasmid transfection was performed using FuGENE HD (Roche, Basel, Switzerland) according to the manufacturer's instructions. 4 µg of DNA was used for each transfection, and transfections were carried out in a six-well plate. For the MXI1 rescue experiment, cells were cotransfected with 100 nmol/L of miRNA mimic and 0.1 µg of plasmid in a 96-well plate. For luciferase reporter assays, U87 cells or 293T cells were plated onto 96-well plates and transfected with 50 ng of luciferase reporter vectors and 150 ng of either pLL3.7-miR-155 or pLL3.7-miR-control vector using FuGENE HD according to the manufacturer's instructions.

### Dual-luciferase reporter assay

The cells were harvested 48 h after transfection and luciferase activity was assessed using the dual-luciferase reporter assay system (Promega, Madison, WI, USA) according to the manufacturer's instructions. *Renilla* luciferase activities were normalized to firefly luciferase activities.

### Western blotting

U87 or 293T cells were lysed with RAPI lysis buffer (BioTeke, Beijing, China) 48 h after transfection. The whole-cell protein concentration was determined by bicinchoninic acid protein assay kit (Beyotime, Shanghai, China). Heat-denatured protein samples (20 µg per lane) were loaded onto a 12% SDS-polyacrylamide gel electrophoresis (SDS-PAGE) and transferred to a PVDF membrane (Millipore, Bedford, MD, USA). The membrane was incubated for 2 h at room temperature with a primary goat polyclonal antibody against human MXI1 (1∶500; Santa Cruz Biotechnology, Santa Cruz, CA, USA) or mouse monoclonal antibody against human β-actin (1∶5000; Abcam, Cambridge, MA, USA) and then incubated for 1 h with a rabbit anti-goat (1∶5000; Abcam, Cambridge, MA, USA) or goat-anti-mouse (1∶10,000; Jackson ImmunoResearch, West Grove, PA, USA) secondary antibody. The bound antibody was detected with the use of enhanced chemiluminescence detection reagents (Pierce, Rockford, IL, USA) according to the manufacturer's instructions. The band intensities were quantified with Kodak Image Station 4000 MM Pro (Kodak, Tokyo, Japan).

### RNA extraction and real-time quantitative reverse transcription-PCR

Total RNA was extracted from cells or tissues using Trizol (Invitrogen, Carlsbad, CA, USA) and reverse transcribed using ReverTra-Ace-α-Transcriptase (TOYOBO Corp., Osaka, Japan). The expression of miR-155 in human tissues was quantified using the SYBR® Premix Ex Taq™ II (Tli RNaseH Plus) kit (Takara Corp., Tokyo, Japan). U6 small nuclear RNA was used as an internal normalized reference. The primers for miR-155 and U6 were purchased from Ribobio, Guangzhou, China. The sequences of the forward and reverse primers to determine MXI1 mRNA levels were 5′-GGAAAAGAATCGACGAGCTCAT-3′ for the forward primer and 5′-GGGTGCAGTCTGGTCCTAGTG-3′ for the reverse primer, with GAPDH as an internal reference. The GAPDH primer sequences were as follows: the forward primer 5′-CCCATGTTCGTCATGGGTGT-3′ and the reverse primer 5′-TGGTCATGAGTCCTTCCACGATA-3′. The quantitative PCR was performed on a LightCycler 480 Real-Time PCR system (Roche, Basel, Switzerland). The data were collected and analyzed using LightCycler 480 Software Version 1.5. The relative fold changes in miRNA or mRNA expression in treated cells vs. control cells or brain cancer tissues vs. normal brain tissues were calculated using the comparative Ct (2^−ΔΔCt^) method [25]. All reactions were carried out in triplicate.

### Cell proliferation assays

Glioma cell proliferation was measured by directly counting cell number, MTT assays and 5-ethynyl-20-deoxyuridine (EdU) incorporation assays. EdU staining was conducted using an EdU assay kit (Ribobio, Guangzhou, China) according to the manufacturers' instructions. To count cell number, 9×10^4^ cells were plated in a 6-well plate, transfected with miRNA mimic or mimic control, and counted after 3 days under a microscope (Nikon, Tokyo, Japan). All experiments were performed in triplicate and three independent repeating experiments were performed. For the MTT assay, one day before transfection, 3×10^3^ U87 cells were seeded into each well of a 96-well plate. 72 h after transfection, MTT reagent (5 mg/ml) was added directly to the medium, and the plate was incubated in a 37°C, 5% CO_2_ incubator for 4 h. The supernatant was removed, and 100 µl of DMSO was added to each well and thoroughly mixed for 10 min. The spectrometric absorbance of the samples at 490 nm was measured on a microplate reader (BioTek, Winooski, USA). All experiments were performed in triplicate and three independent repeating experiments were performed. For the EdU incorporation assay, 4×10^3^ U87 cells were cultured in triplicate in 96-well plates for 24 h at 37°C. 48 h after transfection, 50 µM of EdU was added to each well, and the cells were cultured for additional 2 h at 37°C. The cells were fixed with 4% formaldehyde for 30 min and permeabilized with 0.5% Triton X-100 for 10 min at room temperature. After the wells were washed with PBS three times, 100 µl of 1×Apollo reaction cocktail was added to each well, and the cells were incubated for 30 min at room temperature. The cells were subsequently stained with 100 µl of Hoechst 33342 for 30 min and visualized under an ECLIPSE Ti-U fluorescent microscope (Nikon, Tokyo, Japan). The EdU-positive cells (red cells) were counted using NIS-Elements BR 3.0 software (Nikon, Tokyo, Japan). The EdU incorporation rate was expressed as the ratio of the number of EdU-positive cells (red cells) to the total number of Hoechst 33342-positive cells (blue cells). All experiments were performed in triplicate and three independent repeat experiments were performed.

### Caspase assay

3×10^3^ U87 cells were transfected in 96-well plates. The enzymatic activities of caspase-3 and caspase -7 were measured 48 h after transfection with the Caspase-Glo 3/7 Assay kit according to the manufacturer's protocol (Promega, Madison, WI, USA).

### Statistical analysis

The data are presented as the mean ± standard deviation (±SD) of at least three separate experiments. The data were analyzed using Student's t-tests. *P* values<0.05 was considered significant; *P* values<0.01 were considered to be extremely statistically significant differences. Data analyses were performed with Prism software (version 5.0; GraphPad Software, La Jolla, CA, USA) and Excel software (Microsoft, Redmond, WA, USA).

## Results

### MXI1 is down-regulated and targeted by multiple miRNAs in glioma cells

**Figure 1:**
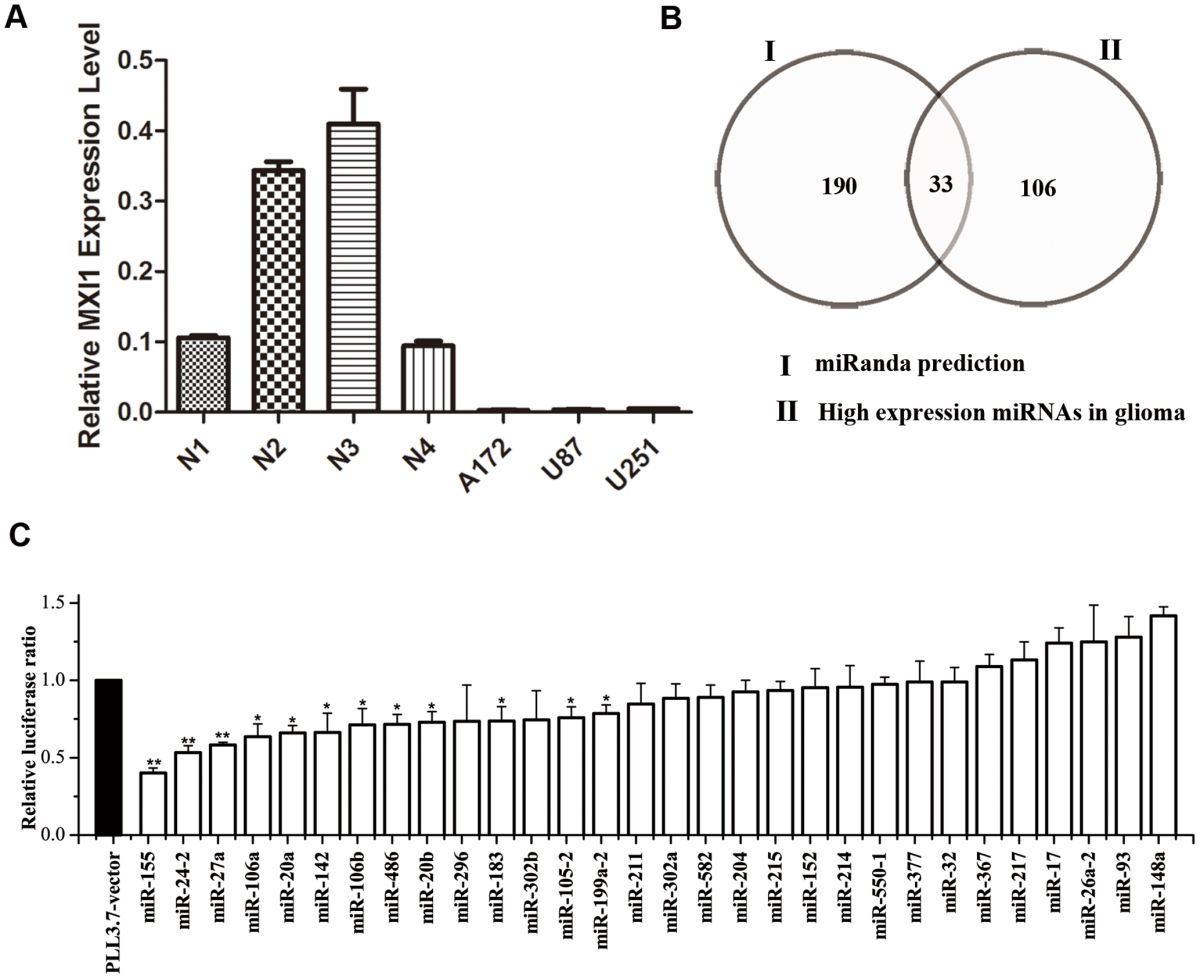
Screening for miRNAs targeting MXI1. (A) qRT-PCR analysis of MXI1 expression level in human normal brain tissues (N1, N2, N3 and N4) and human glioma cell lines (A172, U87 and U251), using GAPDH as internal control. (B) Venn diagram showed the predicted MXI1 3′UTR binding miRNAs that were reported to be up-regulated in gliomas. (C) Effect of 30 miRNAs on the activity of a luciferase reporter gene that is fused with full-length MXI1 3′UTR in 293T cells as tested using a luciferase reporter assay. The data were normalized to the ratio of firefly and Renilla luciferase activities measured at 48 h post-transfection. The results were presented as the relative luciferase activity and normalized to the control, which was assigned a value of 1. The values represent the mean ± SD from three independent transfection experiments. Significant differences from the control value are indicated by * *P*<0.05, ** *P*<0.01.

To evaluate the role of MXI1 in gliomas, we first determined the expression level of MXI1 in A172, U87 and U251 glioma cells by quantitative reverse transcription-PCR (qRT-PCR). The level of MXI1 mRNA is significantly lower in glioma cell lines than the level in human normal brain tissues (Fig. 1A). We hypothesized that some miRNAs, especially up-regulated miRNAs, might be responsible for the low expression of MXI1 in gliomas.

To investigate which miRNAs might target MXI1, we predicted potential MXI1-binding miRNAs using the miRanda algorithm [26] and found that 190 miRNAs could target MXI1. To find the miRNAs that may down-regulate MXI1 under pathogenic conditions, we searched for miRNAs that are highly expressed in gliomas according to eight recently published papers [27]–[34]. We hypothesized that the miRNAs that were reported to be up-regulated in at least two of the eight papers would be more reliable than those reported in one paper. One hundred and six miRNAs were chosen for further analysis using this approach (Fig. 1B). Among the 106 miRNAs, we found 33 miRNAs that were predicted to target MXI1 and up-regulated in gliomas. We then tested 30 (whose precursor expression vectors were available in our miRNA expression library [35]) of these 33 miRNAs using a dual luciferase assay. 12 of these 30 miRNA precursor expression vectors could down-regulate the activity of a luciferase reporter gene that is fused with full-length MXI1 3′UTR in 293T cells (Student's t-test, p<0.05) (Fig. 1C). Among the tested miRNA precursors, the effects of miR-155, miR-24-2 and miR-27a are the most obvious. Because the miRNA precursors of miR-27a and miR-24-2 are located in the miR-23a∼24-2∼27a cluster, we studied the function of this cluster separately [36]. In this study, we focused on miR-155, which is the strongest inhibitor of the reporter gene and is highly expressed in gliomas [30], [32], [33].

### MXI1 is a direct downstream target of miR-155

**Figure 2:**
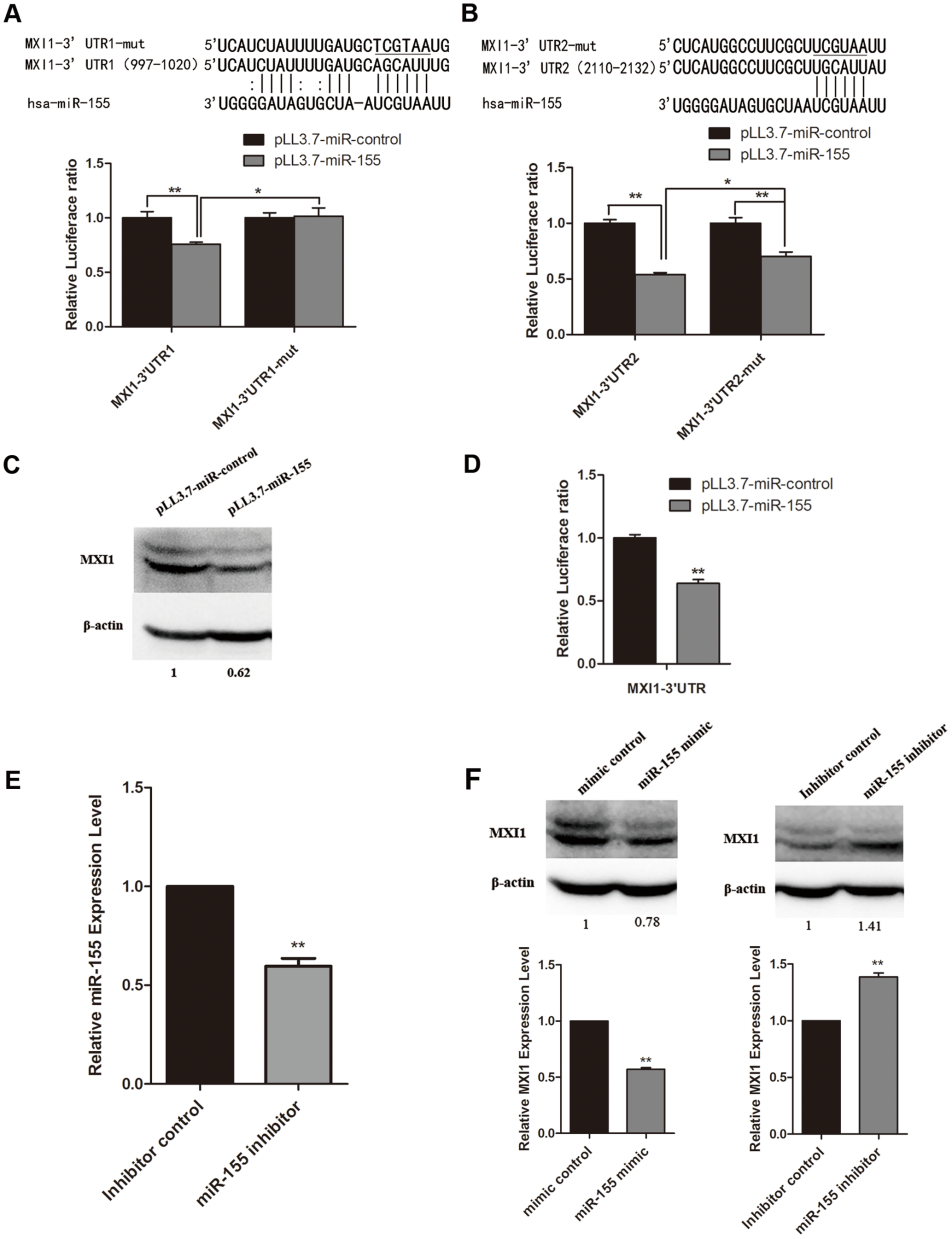
miR-155 down-regulates MXI1 mRNA and protein levels via the MXI1 3′UTR. (A, B) Two predicted target sites of miR-155 within the 3′UTR of MXI1. Several nucleotides within the seed region are mutated in the 3′UTR of MXI1. An empty vector or miRNA expression vector (pre-miR-155) was co-transfected with wild type UTR1, UTR2, or mutant UTR1 and UTR2. The luciferase activity of the transfected cells was measured at 48 h post-transfection. (C) Western blot analysis of MXI1 protein levels after the introduction of miRNA expression vectors (pLL3.7-miR-control or pLL3.7-miR-155) in 293T cells. (D) U87 cells were co-transfected with miR-155 expression vector and full-length MXI1 3′UTR; luciferase activity was measured after 48 h. Each data point was measured in triplicate. The values represent the means ± SD. Significant differences from the control value are indicated by * *P*<0.05, ** *P*<0.01. (E) The relative expression level of miR-155 was measured by qRT-PCR after the introduction of either the inhibitor control or miR-155 inhibitor in U87 cells. U6 served as internal normalized references for miR-155. The average values ± SD of three separate experiments were plotted, ** *P*<0.01. (F) Western blot analysis of MXI1 protein levels after the introduction of mimic control, miR-155 mimic, inhibitor control, or miR-155 inhibitor to U87 cells. The relative expression level of MXI1 was measured by qRT-PCR. β-actin and GAPDH served as internal normalized references for MXI1. The average values ± SD of three separate experiments were plotted, ** *P*<0.01.

Because two target sites for miR-155 exist in the MXI1 3′UTR (one was predicted by miRanda, and the other by microRNA.org), we divided the MXI1 3′UTR into two fragments, MXI1-3′UTR1 and MXI1-3′UTR2; each fragment contains one target site. To determine whether miR-155 recognizes the sites in the 3′UTR of MXI1 mRNA, we cloned the MXI1-3′UTR1, MXI1-3′UTR2, and mutated versions of each (3′UTR1-mut and 3′UTR2-mut) downstream of the *Renilla* luciferase reporter gene in the psiCHECK2 vector to generate the MXI1-3′UTR1, MXI1-3′UTR2, MXI1-3′UTR1-mut and MXI1-3′UTR2-mut vectors (Fig. 2A and B). The MXI1-3′UTR or MXI1-3′UTR-mut vectors were co-transfected with the miR-155 expressing vector pLL3.7-miR-155 into 293T cells, and an empty vector (pLL3.7-miR-control) was used as a control. miR-155 could down-regulate both MXI1-3′UTR1 and MXI1-3′UTR2 in 293T cells. The repressive effect on luciferase activity was abrogated by a mutation in the seed region of the MXI1-3′UTR1 fragment and partially attenuated by a mutation in the seed region of MXI1-3′UTR2 fragment. In addition, Western blot analysis showed that miR-155 substantially down-regulated the level of endogenous Mxi1 protein in 293T cells (Fig. 2C).

The effect of miR-155 on MXI1 was also confirmed in U87 glioma cells. The luciferase activity was decreased significantly when U87 cells were co-transfected with pLL3.7-miR-155 and a luciferase construct containing the full-length MXI1 3′UTR (Fig. 2D). Next, U87 cells were transfected with a synthetic miR-155 mimic and a miR-155 inhibitor. Western blot analysis showed that the level of endogenous Mxi1 protein was reduced by transfection of a miR-155 mimic. In contrast, when the expression of miR-155 in U87 cells was effectively down-regulated by the transfection of a miR-155 inhibitor (Fig. 2E), the level of Mxi1 protein was up-regulated compared to the inhibitor control (Fig. 2F upper panel). Real-time qRT-PCR analysis showed that the level of MXI1 mRNA was changed consistently with protein level (Fig. 2F, lower panel). Taken together, these data suggest that miR-155 directly targets MXI1 via its 3′UTR in 293T and U87 glioma cells.

### miR-155 promotes glioma cell proliferation by the inhibition of MXI1

**Figure 3:**
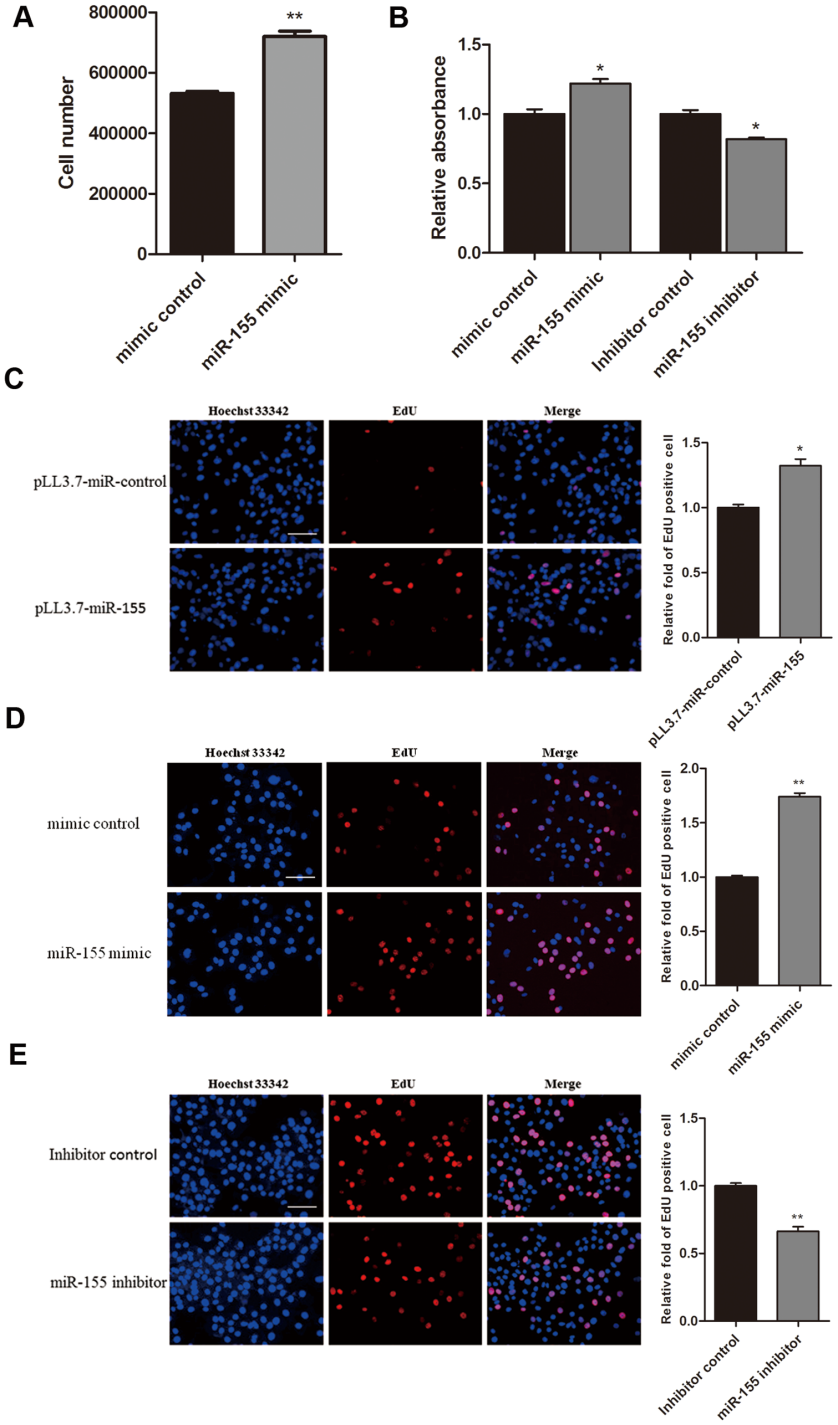
miR-155 promotes cell proliferation in U87 cells. (A) Validation of miR-155 induced proliferation by counting U87 cell number at 72 h after transfection with a miR-155 mimic. (B) U87 cells were transfected with a mimic control, a miR-155 mimic, an inhibitor control, or a miR-155 inhibitor as indicated, and the growth of the cells was investigated using MTT assays after 72 h. (C, D, E) U87 cells were transfected with empty vector, miR-155 expression vector, mimic control, miR-155 mimic, inhibitor control, or miR-155 inhibitor as indicated, and an EdU incorporation assay was carried out (scale bar, 50 µm). The EdU incorporation rate was expressed as the ratio of EdU-positive cells to the total number of Hoechst 33342-positive cells. The average values ± SD of three separate experiments were plotted, * *P*<0.05, ** *P*<0.01.

**Figure 4:**
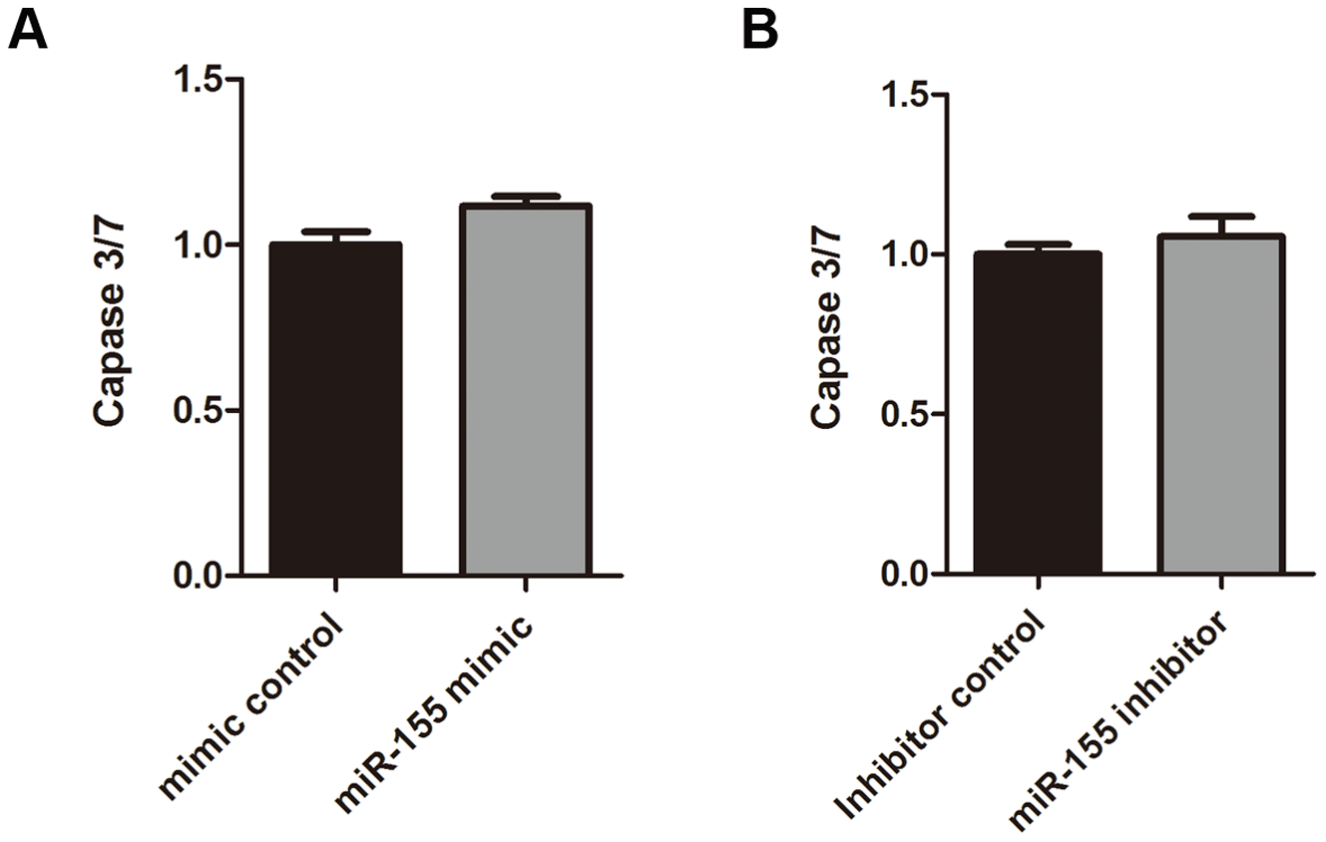
miR-155 has no significant effect on cell apoptosis in U87 cells. Caspase 3/7-Glo assays were performed 72 h after transfection. No statistically significant changes in Caspase 3/7 activity were observed in U87 cells that were treated with a miR-155 mimic (A) or a miR-155 inhibitor (B).

To understand the function of miR-155 in gliomas, we investigated the effect of miR-155 on glioma cell proliferation by directly counting cell number, MTT assays, and EdU assays. As indicated in Figure 3A, the number of U87 cells transfected with the miR-155 mimic was significantly increased compared to the number of cells transfected with the mimic control. The transfection of U87 cells with miR-155 mimic increased the relative absorbance of the cells in an MTT assay. Consistently, transfection of U87 cells with the miR-155 inhibitor decreased relative absorbance (Fig. 3B). Subsequently, we employed the EdU incorporation assay to further determine the function of miR-155 in enhancing U87 cell proliferation. The number of EdU-positive cells was increased by 1.7-fold or 1.3-fold when U87 cells were transfected with miR-155 mimic (Fig. 3C) or pLL3.7-miR-155 vector (Fig. 3D), respectively. In contrast, the number of EdU-positive cells was reduced by 34% in U87 cells that were transfected with the miR-155 inhibitor compared to cells transfected with the inhibitor control (Fig. 3E). Furthermore, a Caspase 3/7-Glo assay showed that miR-155 has no effect on the apoptosis of U87 cells (Fig. 4). These results suggest that the overexpression of miR-155 can enhance U87 cell proliferation and that the inhibition of miR-155 in U87 cells results in a significant reduction in cell proliferation.

**Figure 5:**
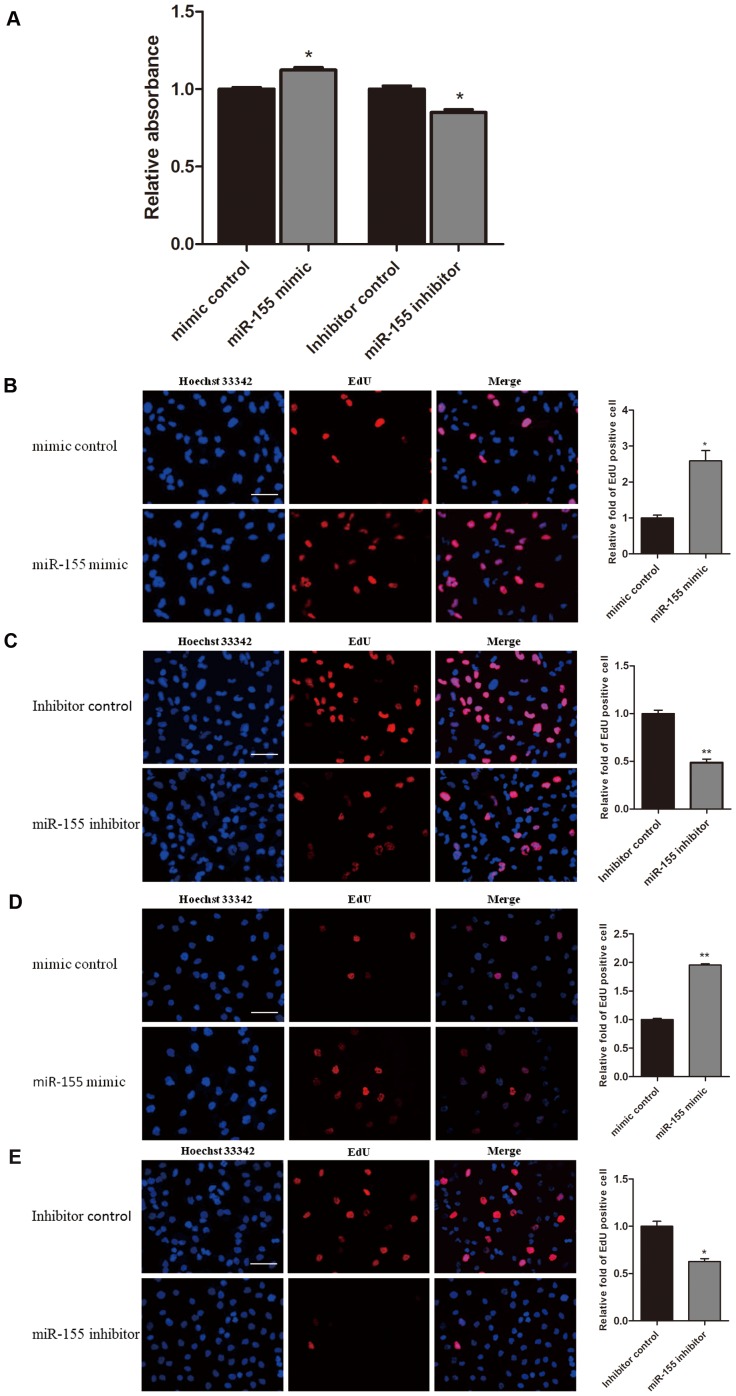
miR-155 promotes cell proliferation in U251 and A172 cells. (A) U251 cells were transfected with a mimic control, a miR-155 mimic, an inhibitor control, or miR-155 inhibitor, and the growth of cell was investigated using an MTT assay after 72 h. The absorbance at 490 nm was normalized to the control to determine cell viability. The given values represent the means ± SD. (B, C) U251 cells were transfected with a mimic control, a miR-155 mimic, an inhibitor control, or a miR-155 inhibitor as indicated, and an EdU incorporation assay was carried out (scale bar, 50 µm). (D, E) A172 cells were transfected with a mimic control, a miR-155 mimic, an inhibitor control, or a miR-155 inhibitor as indicated, and an EdU incorporation assay was carried out (scale bar, 50 µm). The EdU incorporation rate was expressed as the ratio of the number of EdU-positive cells to the total number of Hoechst 33342-positive cells. The average values ± SD of three separate experiments were plotted, * *P*<0.05, ** *P*<0.01.

Similarly, a miR-155 mimic promoted the proliferation of U251 cells, and a miR-155 inhibitor was able to inhibit the proliferation of U251 cells (Fig. 5A). An EdU assay also showed more EdU-positive cells in U251 and A172 glioma cells that were transfected with a miR-155 mimic, respectively (Fig. 5B and D), and fewer EdU-positive cells in U251 and A172 cells that were transfected with a miR-155 inhibitor (Fig. 5C and E). Taken together, these results confirm that miR-155 is capable of promoting glioma cell proliferation.

**Figure 6:**
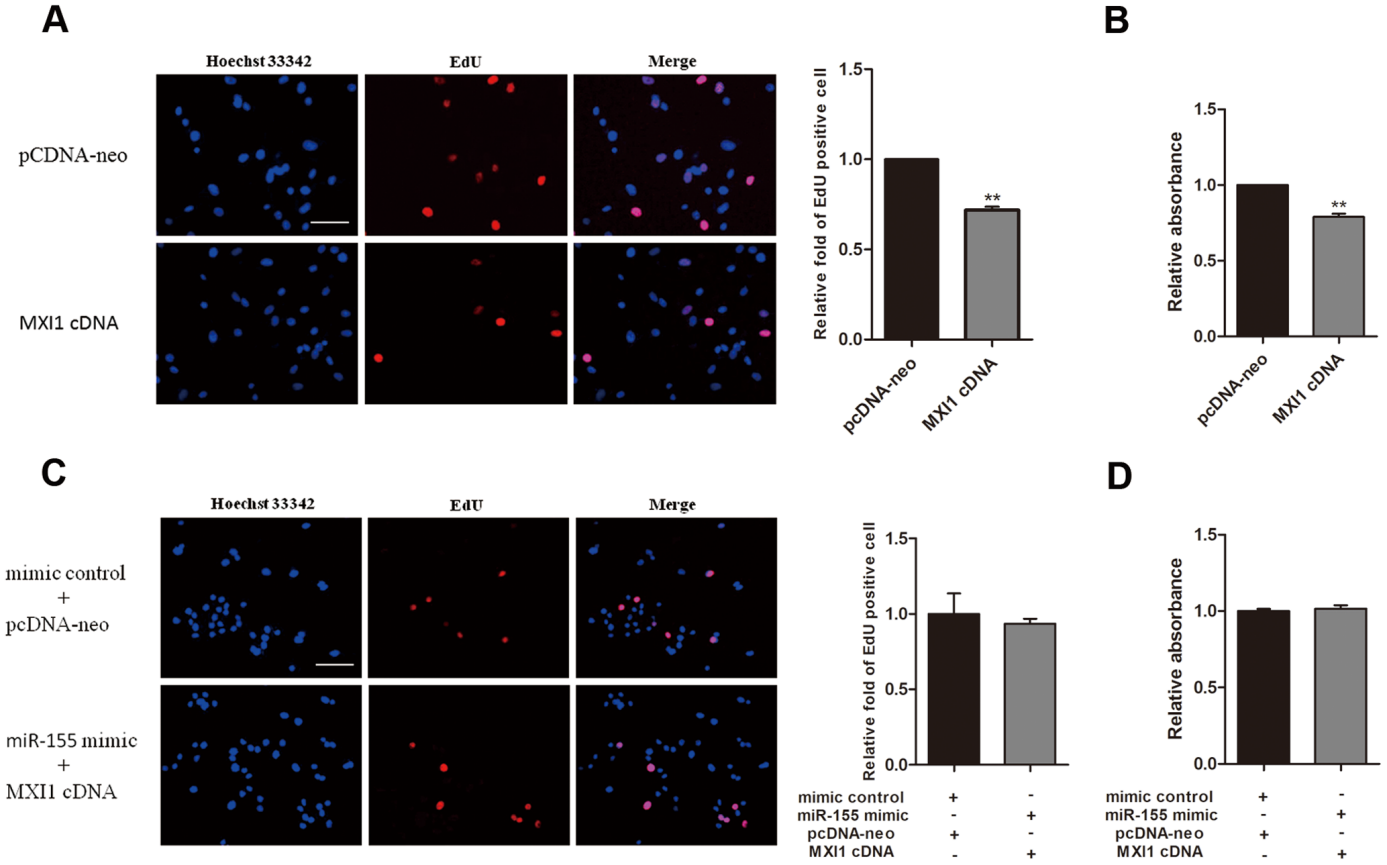
MXI1 cDNA can rescue the effect of miR-155. (A) U87 cells were transfected with empty vector or with MXI1 cDNA, and an EdU incorporation assay was analyzed after 48 h (scale bar, 50 µm). (B) U87 cells were transfected with empty vector or with MXI1 cDNA, and the growth of the cells was investigated using an MTT assay after 72 h. (C) U87 cells were co-transfected with either mimic control and empty vector or miR-155 mimic and MXI1-cDNA. An EdU incorporation assay was analyzed after 48 h (scale bar, 50 µm). The EdU incorporation rate was expressed as the ratio of the number of EdU-positive cells to the total number of Hoechst 33342-positive cells. The average values ± SD of three separate experiments were plotted, ** *P*<0.01. (D) U87 cells were co-transfected with either mimic control and empty vector or miR-155 mimic and MXI1-cDNA, and the cell growth was investigated using an MTT assay after 72 h. The absorbance at 490 nm was normalized to the appropriate control to determine cell viability. Values represent the means ± SD.

To test whether MXI1 mediated the cell proliferation promoted by miR-155 in glioma cells, a MXI1 expression vector was used to perform rescue experiments. The overexpression of MXI1 was validated by Western blot (data not shown), and the proliferation of U87 cells were inhibited as indicated by an EdU incorporation assay (Fig. 6A) and an MTT assay (Fig. 6B). These results were consistent with the previous report [14]. Furthermore, when U87 cells were transfected with both the miR-155 mimic and the MXI1 expression vector, the effect of miR-155 in promoting cell proliferation was significantly attenuated, as indicated by an EdU incorporation assay (Fig. 6C) and an MTT assay (Fig. 6D). These results further suggest that MXI1 is one of the functional downstream targets of miR-155 in promoting glioma cell proliferation.

### The level of MXI1 mRNA is inversely correlated with miR-155 expression in gliomas

**Figure 7:**
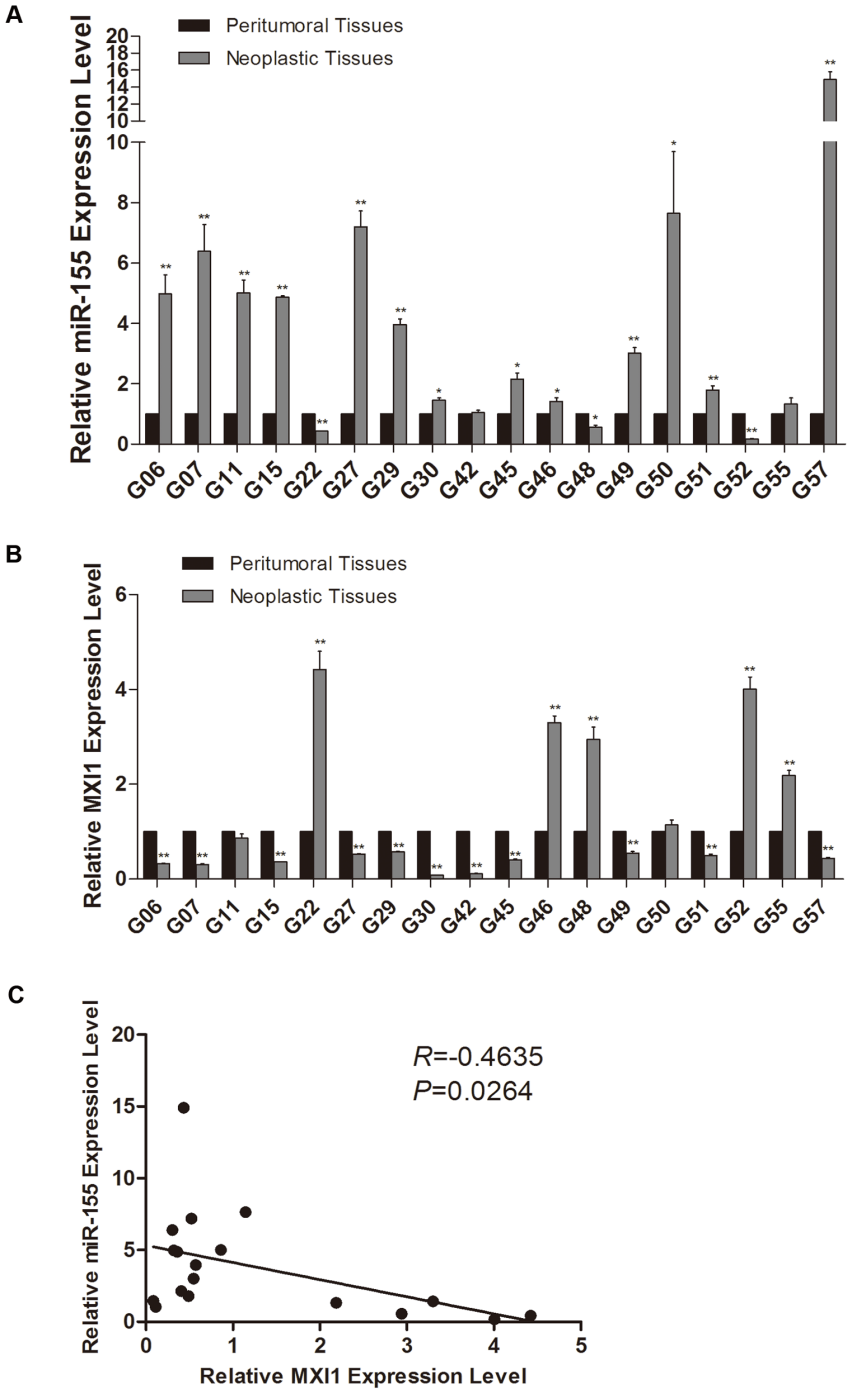
Expression of miR-155 and MXI1 in neoplastic tissue and peritumoral brain tissues. (A, B) The expression of microRNA-155 and MXI1 in 18 pairs of tumor samples (gray bars) was measured relative to that of peritumoral tissues (black bars) using qRT-PCR. GAPDH and U6 served as internal normalized references for MXI1 and miR-155, respectively. The average values ± SD of three separate experiments were plotted, ** *P*<0.01. (C) The association between the miR-155 and MXI1 expression values was evaluated using Pearson rank correlation analysis. The results indicated a statistically significant inverse correlation (*R* = −0.4635, *P* = 0.0264) in these 18 pairs of tumor samples.

Given that MXI1 is functionally targeted by miR-155 in glioma cells, we analyzed the expression level of miR-155 and MXI1 mRNA in 18 sets of glioblastoma multiforme specimens and their corresponding normal adjacent tissues specimens by qRT-PCR. We found that the expression level of miR-155 was significantly higher in approximately 72% of our brain tumor specimens, with an increase of >2-fold in 9 of the 18 tumor pairs (Fig. 7A). This result is consistent with miRNA microarray analysis in gliomas [32]. In addition, we found that MXI1 mRNA levels were reduced by >40% in 61% of brain tumors compared to the corresponding normal brain tissues (Fig. 7B). Using Pearson's correlation analysis of miR-155-MXI1 expression, we obtained a statistically significant inverse correlation (*R* = −0.4635, *P* = 0.0264) in all 18 tumors (Fig. 7C). These results suggest that the expression of miR-155 is inversely correlated with the expression of MXI1 mRNA in gliomas.

## Discussion

MXI1 is considered to be a tumor suppressor gene because it acts as an antagonist of the oncogene c-Myc [3], [4] and negatively regulates the promoter of c-Myc [5]. In this study, we discovered that there is a decreased expression of MXI1 in three different glioma cell lines by qRT-PCR. We have identified for the first time that the MXI1 gene may be directly targeted by several miRNAs, including miR-155. miR-155 is located in the noncoding BIC gene, which was originally identified as a frequent site of integration of the avian leukosis virus [21], [22]. To study the deregulation of MXI1 in gliomas, we initially predicted two miR-155 binding sites in the MXI1 3′UTR using miRanda and microRNA.org (Fig. 2A and B). Then, a dual luciferase reporter assay was performed to confirm the predicted binding sites. miR-155 could significantly down-regulate the activity of luciferase reporter genes via the MXI1 3′UTR. In contrast, when miR-155 binding site 1 in the MXI1 3′ UTR was mutated, the inhibition of luciferase activity was completely abolished, but the inhibition was only attenuated when site 2 was mutated (Fig. 2A and B). This may be because mutations in the seed region are not sufficient to abrogate the interaction between miR-155 and the target site because the sequence in other region also plays a role in the interaction [35] and a large portion of miRNA-mRNA interactions involve the miRNA 3′ end [37]. Additionally, there may be other sites that are targeted by miR-155 and may not be predicted by microRNA target prediction programs. Furthermore, the overexpression of miR-155 suppressed the levels of both MXI1 protein and mRNA, and the down-regulation of miR-155 resulted in the up-regulation of the levels of both MXI1 protein and mRNA. This is consistent with the results showing that MXI1 mRNA was increased by 1.5-fold in miR-155-deficient B cells [38]. All these results suggest that MXI1 is a direct target of miR-155, and this enhances our understanding of the mechanisms of MXI1 down-regulation in glioma cells.

Given the tumor suppressor effect of MXI1, we studied the potential role of miR-155 in glioma cell proliferation by directly counting cell number and performing MTT and EdU incorporation assays. Our experiments in U87, U251 and A172 cells showed that miR-155 positively affected glioma cell proliferation. The overexpression of MXI1 in U87 cells could decrease the effect of miR-155. Therefore, miR-155 may promote the proliferation of glioma cells by directly targeting MXI1. It has been observed that miR-155 and c-myc are always highly expressed in B cell lymphomas [39], [40]. The fact that Mxi1 can negatively regulate c-myc expression via the c-myc promoter [5] may be the reason that miR-155 is positively correlated with c-myc. The co-expression of miR-155 with c-myc has been found to synergize lymphomageneis. Therefore, it is possible that mir-155 functions as an oncogene in cooperation with c-myc [39]–[42].

To further understand the role of miR-155, we quantified the expression of miR-155 and MXI1 in 18 sets of glioblastoma multiforme specimens and their corresponding normal adjacent tissues. Elevated miR-155 expression occurred in 72% of our brain tumor specimens (Fig. 7A). Recently, increasing numbers of studies have revealed that miR-155 plays important roles in mammalian physiological processes that involve innate and adaptive immunity, viral infection and oncogenesis [43]. This may partially explain why miR-155 is up-regulated in many types of tumors, including lymphoma, breast, lung, pancreatic, thyroid cancer and gliomas [21], [32], [44]–[47]. However, the function and the mechanism of the highly expressed miR-155 in glioma have not been fully determined. In this study, we found that the expression of MXI1 mRNA in 18 pairs of samples was reduced by >40% in 61% of our brain tumor samples. The expression of miR-155 and MXI1 in glioma samples was analyzed and showed a significant inverse correlation. This finding further confirmed that miR-155 could target MXI1 in glioma samples.

In summary, from screening a panel of up-regulated miRNAs in glioma, miR-155 was found strongly to target MXI1, which is a tumor suppressor gene that is down-regulated in gliomas. By increasing the level of miR-155 expression, it was revealed that miR-155 stimulates glioma cell proliferation. Furthermore, the overexpression of the MXI1 gene could rescue the effect of miR-155 on cell proliferation. Mutations in the miR-155 targeting sites in MXI1 abrogated or attenuated this inhibitory effect. At the same time, we determined that the level of MXI1 mRNA was inversely correlated with miR-155 expression in glioblastoma multiforme specimens. This result suggests that miR-155 directly and functionally targets MXI1, which may relate to glioma-genesis or glioma progression. Exploiting miR-155-based therapy may be beneficial for the clinical treatment of cancers, including gliomas.

## Supporting Information

Table S1 The primers of pre-miRNAs used in this study.
(DOC)

## References

[1] Zhang Y, Dutta A, Abounader R (2012) The role of microRNAs in glioma initiation and progression. Front Biosci (Landmark Ed) 17: 700–712. 22201769 10.2741/3952 PMC3278211

[2] Xu X, Xi L, Zeng J, Yao Q (2012) A Functional+61G/A Polymorphism in Epidermal Growth Factor Is Associated with Glioma Risk among Asians. PLoS One 7: e41470. 22829952 10.1371/journal.pone.0041470 PMC3400669

[3] Schreiber-Agus N, Chin L, Chen K, Torres R, Rao G, et al (1995) An amino-terminal domain of Mxi1 mediates anti-Myc oncogenic activity and interacts with a homolog of the yeast transcriptional repressor SIN3. Cell 80: 777–786. 7889571 10.1016/0092-8674(95)90356-9

[4] Laherty CD, Yang WM, Sun JM, Davie JR, Seto E, et al (1997) Histone deacetylases associated with the mSin3 corepressor mediate mad transcriptional repression. Cell 89: 349–356. 9150134 10.1016/s0092-8674(00)80215-9

[5] Lee TC, Ziff EB (1999) Mxi1 is a repressor of the c-Myc promoter and reverses activation by USF. Journal of Biological Chemistry 274: 595–606. 9872993 10.1074/jbc.274.2.595

[6] Schreiber-Agus N, Meng Y, Hoang T, Hou H, Chen K, et al (1998) Role of Mxi1 in ageing organ systems and the regulation of normal and neoplastic growth. Nature 393: 483–487. 9624006 10.1038/31008

[7] Wechsler DS, Hawkins AL, Li X (1994) Localization of the human Mxi1 transcription factor gene (MXl1) to chromosome 10q24-q25. Genomics 21: 669–672. 7959753 10.1006/geno.1994.1336

[8] Edelhoff S, Ayer DE, Zervos AS, Steingrimsson E, Jenkins N, et al (1994) Mapping of two genes encoding members of a distinct subfamily of MAX interacting proteins: MAD to human chromosome 2 and mouse chromosome 6, and MXII to human chromosome 10 and mouse chromosome 19. Oncogene-Basingstoke 9: 665–665. 8290278

[9] Wang DY, Xiang YY, Li XJ, Hashimoto M, Tanaka M, et al (2000) Mxi1 is a potential cellular target of carcinogens and frequently mutated in experimental rat tumors and tumor cell lines. Pathology International 50: 373–383. 10849326 10.1046/j.1440-1827.2000.01057.x

[10] Prochownik EV, Grove LE, Deubler D, Zhu XL, Stephenson RA, et al (1998) Commonly occurring loss and mutation of the MXI1 gene in prostate cancer. Genes, Chromosomes and Cancer 22: 295–304. 9669667

[11] Eagle LR, Yin X, Brothman AR, Williams BJ, Atkin N, et al (1995) Mutation of the MXI1 gene in prostate cancer. Nature genetics 9: 249–255. 7773287 10.1038/ng0395-249

[12] Li XJ, Wang DY, Zhu Y, Guo RJ, Wang XD, et al (1999) Mxi1 mutations in human neurofibrosarcomas. Cancer Science 90: 740–746. 10.1111/j.1349-7006.1999.tb00809.x PMC5926139 10470286

[13] Kawamata N, Park D, Wilczynski S, Yokota J, Koeffler HP (2007) Point mutations of the Mxil gene are rare in prostate cancers. The Prostate 29: 191–193. 10.1002/1097-0045(199609)29:3<191::aid-pros2990290305>3.0.co;2-1 8827088

[14] Wechsler DS, Shelly CA, Petroff CA, Dang CV (1997) MXI1, a putative tumor suppressor gene, suppresses growth of human glioblastoma cells. Cancer research 57: 4905–4912. 9354456

[15] Fults D, Pedone C, Thompson G, Uchiyama C, Gumpper K, et al (1998) Microsatellite deletion mapping on chromosome 10q and mutation analysis of MMAC1, FAS, and MXI1 in human glioblastoma multiforme. Int J Oncol 12: 905. 9499454 10.3892/ijo.12.4.905

[16] Skotheim RI, Monni O, Mousses S, Fossa SD, Kallioniemi OP, et al (2002) New insights into testicular germ cell tumorigenesis from gene expression profiling. Cancer Res 62: 2359–2364. 11956097

[17] Manni I, Tunici P, Cirenei N, Albarosa R, Colombo B, et al (2002) Mxi1 inhibits the proliferation of U87 glioma cells through down-regulation of cyclin B1 gene expression. British Journal of Cancer 86: 477–484. 11875718 10.1038/sj.bjc.6600065 PMC2375210

[18] Eulalio A, Huntzinger E, Izaurralde E (2008) Getting to the root of miRNA-mediated gene silencing. Cell 132: 9–14. 18191211 10.1016/j.cell.2007.12.024

[19] Lujambio A, Lowe SW (2012) The microcosmos of cancer. Nature 482: 347–355. 22337054 10.1038/nature10888 PMC3509753

[20] Rodriguez A, Vigorito E, Clare S, Warren MV, Couttet P, et al (2007) Requirement of bic/microRNA-155 for normal immune function. Science 316: 608–611. 17463290 10.1126/science.1139253 PMC2610435

[21] Kluiver J, Poppema S, de Jong D, Blokzijl T, Harms G, et al (2005) BIC and miR-155 are highly expressed in Hodgkin, primary mediastinal and diffuse large B cell lymphomas. J Pathol 207: 243–249. 16041695 10.1002/path.1825

[22] Lagos-Quintana M, Rauhut R, Yalcin A, Meyer J, Lendeckel W, et al (2002) Identification of tissue-specific microRNAs from mouse. Curr Biol 12: 735–739. 12007417 10.1016/s0960-9822(02)00809-6

[23] Tam W (2001) Identification and characterization of human BIC, a gene on chromosome 21 that encodes a noncoding RNA. Gene 274: 157–167. 11675008 10.1016/s0378-1119(01)00612-6

[24] Eis PS, Tam W, Sun L, Chadburn A, Li Z, et al (2005) Accumulation of miR-155 and BIC RNA in human B cell lymphomas. Proc Natl Acad Sci U S A 102: 3627–3632. 15738415 10.1073/pnas.0500613102 PMC552785

[25] Livak KJ, Schmittgen TD (2001) Analysis of relative gene expression data using real-time quantitative PCR and the 2(-Delta Delta C(T)) Method. Methods 25: 402–408. 11846609 10.1006/meth.2001.1262

[26] John B, Enright AJ, Aravin A, Tuschl T, Sander C, et al (2004) Human MicroRNA targets. PLoS Biol 2: e363. 15502875 10.1371/journal.pbio.0020363 PMC521178

[27] Ciafre SA, Galardi S, Mangiola A, Ferracin M, Liu CG, et al (2005) Extensive modulation of a set of microRNAs in primary glioblastoma. Biochem Biophys Res Commun 334: 1351–1358. 16039986 10.1016/j.bbrc.2005.07.030

[28] Godlewski J, Nowicki MO, Bronisz A, Williams S, Otsuki A, et al (2008) Targeting of the Bmi-1 oncogene/stem cell renewal factor by microRNA-128 inhibits glioma proliferation and self-renewal. Cancer Res 68: 9125–9130. 19010882 10.1158/0008-5472.CAN-08-2629

[29] Zhou X, Ren Y, Moore L, Mei M, You Y, et al (2010) Downregulation of miR-21 inhibits EGFR pathway and suppresses the growth of human glioblastoma cells independent of PTEN status. Lab Invest 90: 144–155. 20048743 10.1038/labinvest.2009.126

[30] Rao SA, Santosh V, Somasundaram K (2010) Genome-wide expression profiling identifies deregulated miRNAs in malignant astrocytoma. Mod Pathol 23: 1404–1417. 20711171 10.1038/modpathol.2010.135

[31] Lavon I, Zrihan D, Granit A, Einstein O, Fainstein N, et al (2010) Gliomas display a microRNA expression profile reminiscent of neural precursor cells. Neuro Oncol 12: 422–433. 20406893 10.1093/neuonc/nop061 PMC2940621

[32] Lages E, Guttin A, El Atifi M, Ramus C, Ipas H, et al (2011) MicroRNA and target protein patterns reveal physiopathological features of glioma subtypes. PLoS One 6: e20600. 21655185 10.1371/journal.pone.0020600 PMC3105101

[33] Skalsky RL, Cullen BR (2011) Reduced expression of brain-enriched microRNAs in glioblastomas permits targeted regulation of a cell death gene. PLoS One 6: e24248. 21912681 10.1371/journal.pone.0024248 PMC3166303

[34] Wuchty S, Arjona D, Li A, Kotliarov Y, Walling J, et al (2011) Prediction of Associations between microRNAs and Gene Expression in Glioma Biology. PLoS One 6: e14681. 21358821 10.1371/journal.pone.0014681 PMC3040173

[35] Zhou P, Xu W, Peng X, Luo Z, Xing Q, et al (2013) Large-Scale Screens of miRNA-mRNA Interactions Unveiled That the 3′UTR of a Gene Is Targeted by Multiple miRNAs. PLoS One 8: e68204. 23874542 10.1371/journal.pone.0068204 PMC3706477

[36] Xu W, Liu M, Peng X, Zhou P, Zhou J, et al (2013) miR-24-3p and miR-27a-3p promote cell proliferation in glioma cells via cooperative regulation of MXI1. Int J Oncol 42: 757–766. 23254855 10.3892/ijo.2012.1742

[37] Helwak A, Kudla G, Dudnakova T, Tollervey D (2013) Mapping the human miRNA interactome by CLASH reveals frequent noncanonical binding. Cell 153: 654–665. 23622248 10.1016/j.cell.2013.03.043 PMC3650559

[38] Vigorito E, Perks KL, Abreu-Goodger C, Bunting S, Xiang Z, et al (2007) microRNA-155 regulates the generation of immunoglobulin class-switched plasma cells. Immunity 27: 847–859. 18055230 10.1016/j.immuni.2007.10.009 PMC4135426

[39] Esquela-Kerscher A, Slack FJ (2006) Oncomirs - microRNAs with a role in cancer. Nat Rev Cancer 6: 259–269. 16557279 10.1038/nrc1840

[40] Tam W, Ben-Yehuda D, Hayward WS (1997) bic, a novel gene activated by proviral insertions in avian leukosis virus-induced lymphomas, is likely to function through its noncoding RNA. Mol Cell Biol 17: 1490–1502. 9032277 10.1128/mcb.17.3.1490 PMC231875

[41] Clurman B, Hayward W (1989) Multiple proto-oncogene activations in avian leukosis virus-induced lymphomas: evidence for stage-specific events. Mol Cell Biol 9: 2657–2664. 2548084 10.1128/mcb.9.6.2657-2664.1989 PMC362338

[42] Tam W, Hughes SH, Hayward WS, Besmer P (2002) Avian bic, a gene isolated from a common retroviral site in avian leukosis virus-induced lymphomas that encodes a noncoding RNA, cooperates with c-myc in lymphomagenesis and erythroleukemogenesis. J Virol 76: 4275–4286. 11932393 10.1128/JVI.76.9.4275-4286.2002 PMC155062

[43] Teng G, Papavasiliou FN (2009) Shhh! Silencing by microRNA-155. Philos Trans R Soc Lond B Biol Sci 364: 631–637. 19008191 10.1098/rstb.2008.0209 PMC2660923

[44] Iorio MV, Ferracin M, Liu CG, Veronese A, Spizzo R, et al (2005) MicroRNA gene expression deregulation in human breast cancer. Cancer Res 65: 7065–7070. 16103053 10.1158/0008-5472.CAN-05-1783

[45] Yanaihara N, Caplen N, Bowman E, Seike M, Kumamoto K, et al (2006) Unique microRNA molecular profiles in lung cancer diagnosis and prognosis. Cancer Cell 9: 189–198. 16530703 10.1016/j.ccr.2006.01.025

[46] Greither T, Grochola LF, Udelnow A, Lautenschlager C, Wurl P, et al (2010) Elevated expression of microRNAs 155, 203, 210 and 222 in pancreatic tumors is associated with poorer survival. Int J Cancer 126: 73–80. 19551852 10.1002/ijc.24687

[47] Nikiforova MN, Tseng GC, Steward D, Diorio D, Nikiforov YE (2008) MicroRNA expression profiling of thyroid tumors: biological significance and diagnostic utility. J Clin Endocrinol Metab 93: 1600–1608. 18270258 10.1210/jc.2007-2696 PMC2386678

