# Supplementary material for: MicroRNA-155 Promotes Glioma Cell Proliferation via the Regulation of MXI1
Source: PLoS One. 2013 Dec 23;8(12):e83055. doi: 10.1371/journal.pone.0083055 (PMC3871643; doi:10.1371/journal.pone.0083055)
Supplement: Table S1 — The primers of pre-miRNAs used in this study. (DOC) [file pone.0083055.s001.doc]

Table S1. The primers of pre-miRNAs used in this study

| **Pre-miRNAs** | **Forward primer sequence( 5' to 3')** | **Reverse primer sequence( 5' to 3')** |
| --- | --- | --- |
| [miR-155](http://microrna.sanger.ac.uk/cgi-bin/sequences/mirna_entry.pl?acc=MI0000681) | CACAACTCGAGCACTCCAGCTTTATAACCGCATGT | AAGGATCCGAACATCCCAGTGACCAGATTATGA |
| [miR-24-2](http://microrna.sanger.ac.uk/cgi-bin/sequences/mirna_entry.pl?acc=MI0000081) | CCGCTCGAGCCTCACCTCCTCTGGCCTTG | CTCCTCCAGACACAGGTCTGATT |
| [miR-27a](http://microrna.sanger.ac.uk/cgi-bin/sequences/mirna_entry.pl?acc=MI0000085) | CACAAGTCGACCTGAGCTCTGCCACCGAGGATGCT | AAGAATCCCAGGCAGCAGGATGGCAGGCAGAC |
| [miR-106a](http://microrna.sanger.ac.uk/cgi-bin/sequences/mirna_entry.pl?acc=MI0000113) | CACAAGTCGACTGTTTTGTTGTTTTAACCAGGTGAG | AAGGATCCGGCATTTAGGGCAGTAGATTCTAAG |
| [miR-20a](http://microrna.sanger.ac.uk/cgi-bin/sequences/mirna_entry.pl?acc=MI0000076) | CACAACTCGAGGTGGCCTGCTATTTCCTTCAAAT | AAGGATCCTTTTCACTACCACAGTCAGTTTTGC |
| [miR-142](http://microrna.sanger.ac.uk/cgi-bin/sequences/mirna_entry.pl?acc=MI0000458) | CACAACTCGAGGGATCTTAGGAAGCCACAAGGAG | AAGGATCCATGGAGGCCTTTCAGGCATCTG |
| [miR-106b](http://microrna.sanger.ac.uk/cgi-bin/sequences/mirna_entry.pl?acc=MI0000734) | CCGCTCGAGACTGCTCTGGTAAGTGCCCAAAT | GGAGGACAGAAAGGAAGGTCTGTA |
| [miR-486](http://microrna.sanger.ac.uk/cgi-bin/sequences/mirna_entry.pl?acc=MI0002470) | CACAAGTCGACTGCCATGAGGAAGGGACATGAAGAC | AAGGATCCTGATTTTTGCCGAATGCAGAAAG |
| [miR-20b](http://microrna.sanger.ac.uk/cgi-bin/sequences/mirna_entry.pl?acc=MI0001519) | CACAACTCGAGTACAGCATTTTAAAAGTATGCCTTGAG | AAGGATCCTGAAATGCAAACCTGCAAAACTAA |
| [miR-296](http://microrna.sanger.ac.uk/cgi-bin/sequences/mirna_entry.pl?acc=MI0000747) | CACAACTCGAGAGAGGGCCTGTGTCTGTGGATCT | AAGGATCCGGCGAGTTCACCATTCCCCAGTGC |
| [miR-183](http://microrna.sanger.ac.uk/cgi-bin/sequences/mirna_entry.pl?acc=MI0000273) | CACAAGTCGACAGCAGCCGCTGCTGAGGGCCTGCT | AAGGATCCGGGCTCTCTGGGGACACACTGGAC |
| [miR-302b](http://microrna.sanger.ac.uk/cgi-bin/sequences/mirna_entry.pl?acc=MI0000772) | CACAACTCGAGCCTTTTACCCTTCTGGAGGAGAAC | TGAAGATCTGGAGACACCTCCACTGAAACAT |
| [miR-105-2](http://microrna.sanger.ac.uk/cgi-bin/sequences/mirna_entry.pl?acc=MI0000112) | CACAAGTCGACTTGTACTGGGAATGAGTATTTTGT | AAGGATCCTTGGCTCCACTATCCATTATTGTA |
| [miR-199a-2](http://microrna.sanger.ac.uk/cgi-bin/sequences/mirna_entry.pl?acc=MI0000281) | CACAAGTCGACTGGTCCTAAATTCATTGCCAGTTC | AAGGATCCCTTCCCTTCGGCAGTCTTTTCTCA |
| [miR-211](http://microrna.sanger.ac.uk/cgi-bin/sequences/mirna_entry.pl?acc=MI0000287) | CACAACTCGAGGGATGGCCTACTAGGAAAAGTTG | AAGGATCCAACCCAAGCAAGGATTGATATTT |
| [miR-302a](http://microrna.sanger.ac.uk/cgi-bin/sequences/mirna_entry.pl?acc=MI0000738) | CACAACTCGAGTTCCATGTTTCAGTGGAGGTGTCT | AAGGATCCGGGCCCCTTAACAGATGTAAAAAT |
| [miR-582](http://microrna.sanger.ac.uk/cgi-bin/sequences/mirna_entry.pl?acc=MI0003589) | CACAACTCGAGTATGTTGCTTCAAGTCATTCATGC | AAGGATCCTTGATACAGCATTACAAATTGATGA |
| [miR-204](http://microrna.sanger.ac.uk/cgi-bin/sequences/mirna_entry.pl?acc=MI0000284) | CACAAGTCGACAAGAGGACCTCCTGATCGTGTATC | AAGGATCCGGTTTGGACCCAGAACTATTAGTCT |
| [miR-215](http://microrna.sanger.ac.uk/cgi-bin/sequences/mirna_entry.pl?acc=MI0000291) | CACAAGTCGACTCTCATTTGATTCCAGCAGAAAAA | AAGGATCCTGAGCTTTGTCAGAAAAACACAAG |
| [miR-152](http://microrna.sanger.ac.uk/cgi-bin/sequences/mirna_entry.pl?acc=MI0000462) | CCGCTCGAGGAGGGTAAGGAGTGGCCTGT | ACTCCCAGGCAGATGTTCCACT |
| [miR-214](http://microrna.sanger.ac.uk/cgi-bin/sequences/mirna_entry.pl?acc=MI0000290) | CCGCTCGAGATTATATTGTGTATTTTTCTCCCTTTCC | GAGCCCCTCATTTTGGTTGTAG |
| [miR-550-1](http://microrna.sanger.ac.uk/cgi-bin/sequences/mirna_entry.pl?acc=MI0003600) | CACAACTCGAGGAAGTTTCTGCTTTGGGAATTTT | AAGGATCCGGCAATTCTCCTTAAAACCCAAATA |
| [miR-377](http://microrna.sanger.ac.uk/cgi-bin/sequences/mirna_entry.pl?acc=MI0000785) | CACAAGTCGACAGCTGGAGTCAGCAGGGAGGTC | AAGGATCCATCTGTTCTCATCCACCGCAGAG |
| [miR-32](http://microrna.sanger.ac.uk/cgi-bin/sequences/mirna_entry.pl?acc=MI0000090) | CACAACTCGAGCTTTATCCCCAGACCATTTTCCTC | AAGGATCCTGTTCACTTTCCAAATGAGAATCG |
| [miR-367](http://microrna.sanger.ac.uk/cgi-bin/sequences/mirna_entry.pl?acc=MI0000775) | CACAAGTCGACTTTAACATGGAGGCACTTGCTG | AAGGATCCAAAGATAGCTCTCTCTTGAAAACA |
| [miR-217](http://microrna.sanger.ac.uk/cgi-bin/sequences/mirna_entry.pl?acc=MI0000293) | CACAACTCGAGTGGGAAAATTATTTGTTAGTATGACTT | AAGGATCCGTTTTAGCATCTTGGGCTCACCT |
| [miR-17](http://microrna.sanger.ac.uk/cgi-bin/sequences/mirna_entry.pl?acc=MI0000071) | CACAACTCGAGCTTCCCCATTAGGGATTATGCTGA | AAGGATCCTTATGCCAGAAGGAGCACTTAGGG |
| [miR-26a-2](http://microrna.sanger.ac.uk/cgi-bin/sequences/mirna_entry.pl?acc=MI0000750) | CACAAGTCGACAGATCGGGTGCAGAGCAAGACT | AAGGATCCGGAGAACTGGCTGTACCCCTCAC |
| [miR-93](http://microrna.sanger.ac.uk/cgi-bin/sequences/mirna_entry.pl?acc=MI0000095) | CCGCTCGAGTTTTCCCCACTTCTTAACCTTCAC | GTGAGGGAGACCAGACCCTTTT |
| [miR-148a](http://microrna.sanger.ac.uk/cgi-bin/sequences/mirna_entry.pl?acc=MI0000253) | CCGCTCGAGTATCGGTCGCATCCTGAACTAAAT | AAGGATCCGTGCTGCACTTGGACCCCCTCTG |
